# Initial Severity and Antidepressant Benefits: A Meta-Analysis of Data Submitted to the Food and Drug Administration

**DOI:** 10.1371/journal.pmed.0050045

**Published:** 2008-02-26

**Authors:** Irving Kirsch, Brett J Deacon, Tania B Huedo-Medina, Alan Scoboria, Thomas J Moore, Blair T Johnson

**Affiliations:** 1 Department of Psychology, University of Hull, Hull, United Kingdom; 2 University of Wyoming, Laramie, Wyoming, United States of America; 3 Center for Health, Intervention, and Prevention, University of Connecticut, Storrs, Connecticut, United States of America; 4 Department of Psychology, University of Windsor, Windsor, Ontario, Canada; 5 Institute for Safe Medication Practices, Huntingdon Valley, Pennsylvania, United States of America; University of Western Sydney, Australia

## Abstract

**Background:**

Meta-analyses of antidepressant medications have reported only modest benefits over placebo treatment, and when unpublished trial data are included, the benefit falls below accepted criteria for clinical significance. Yet, the efficacy of the antidepressants may also depend on the severity of initial depression scores. The purpose of this analysis is to establish the relation of baseline severity and antidepressant efficacy using a relevant dataset of published and unpublished clinical trials.

**Methods and Findings:**

We obtained data on all clinical trials submitted to the US Food and Drug Administration (FDA) for the licensing of the four new-generation antidepressants for which full datasets were available. We then used meta-analytic techniques to assess linear and quadratic effects of initial severity on improvement scores for drug and placebo groups and on drug–placebo difference scores. Drug–placebo differences increased as a function of initial severity, rising from virtually no difference at moderate levels of initial depression to a relatively small difference for patients with very severe depression, reaching conventional criteria for clinical significance only for patients at the upper end of the very severely depressed category. Meta-regression analyses indicated that the relation of baseline severity and improvement was curvilinear in drug groups and showed a strong, negative linear component in placebo groups.

**Conclusions:**

Drug–placebo differences in antidepressant efficacy increase as a function of baseline severity, but are relatively small even for severely depressed patients. The relationship between initial severity and antidepressant efficacy is attributable to decreased responsiveness to placebo among very severely depressed patients, rather than to increased responsiveness to medication.

## Abstract 2

Kirsch and colleagues show that, in antidepressant trials, there is a greater difference in efficacy between drug and placebo amongst more severely depressed patients. However, this difference seems to result from a poorer response to placebo amongst more depressed patients.

## Editors' Summary

**Background.:**

Everyone feels miserable occasionally. But for some people—those with depression—these sad feelings last for months or years and interfere with daily life. Depression is a serious medical illness caused by imbalances in the brain chemicals that regulate mood. It affects one in six people at some time during their life, making them feel hopeless, worthless, unmotivated, even suicidal. Doctors measure the severity of depression using the “Hamilton Rating Scale of Depression” (HRSD), a 17–21 item questionnaire. The answers to each question are given a score and a total score for the questionnaire of more than 18 indicates severe depression. Mild depression is often treated with psychotherapy or talk therapy (for example, cognitive–behavioral therapy helps people to change negative ways of thinking and behaving). For more severe depression, current treatment is usually a combination of psychotherapy and an antidepressant drug, which is hypothesized to normalize the brain chemicals that affect mood. Antidepressants include “tricyclics,” “monoamine oxidases,” and “selective serotonin reuptake inhibitors” (SSRIs). SSRIs are the newest antidepressants and include fluoxetine, venlafaxine, nefazodone, and paroxetine.

**Why Was This Study Done?:**

Although the US Food and Drug Administration (FDA), the UK National Institute for Health and Clinical Excellence (NICE), and other licensing authorities have approved SSRIs for the treatment of depression, some doubts remain about their clinical efficacy. Before an antidepressant is approved for use in patients, it must undergo clinical trials that compare its ability to improve the HRSD scores of patients with that of a placebo, a dummy tablet that contains no drug. Each individual trial provides some information about the new drug's effectiveness but additional information can be gained by combining the results of all the trials in a “meta-analysis,” a statistical method for combining the results of many studies. A previously published meta-analysis of the published and unpublished trials on SSRIs submitted to the FDA during licensing has indicated that these drugs have only a marginal clinical benefit. On average, the SSRIs improved the HRSD score of patients by 1.8 points more than the placebo, whereas NICE has defined a significant clinical benefit for antidepressants as a drug–placebo difference in the improvement of the HRSD score of 3 points. However, average improvement scores may obscure beneficial effects between different groups of patient, so in the meta-analysis in this paper, the researchers investigated whether the baseline severity of depression affects antidepressant efficacy.

**What Did the Researchers Do and Find?:**

The researchers obtained data on all the clinical trials submitted to the FDA for the licensing of fluoxetine, venlafaxine, nefazodone, and paroxetine. They then used meta-analytic techniques to investigate whether the initial severity of depression affected the HRSD improvement scores for the drug and placebo groups in these trials. They confirmed first that the overall effect of these new generation of antidepressants was below the recommended criteria for clinical significance. Then they showed that there was virtually no difference in the improvement scores for drug and placebo in patients with moderate depression and only a small and clinically insignificant difference among patients with very severe depression. The difference in improvement between the antidepressant and placebo reached clinical significance, however, in patients with initial HRSD scores of more than 28—that is, in the most severely depressed patients. Additional analyses indicated that the apparent clinical effectiveness of the antidepressants among these most severely depressed patients reflected a decreased responsiveness to placebo rather than an increased responsiveness to antidepressants.

**What Do These Findings Mean?:**

These findings suggest that, compared with placebo, the new-generation antidepressants do not produce clinically significant improvements in depression in patients who initially have moderate or even very severe depression, but show significant effects only in the most severely depressed patients. The findings also show that the effect for these patients seems to be due to decreased responsiveness to placebo, rather than increased responsiveness to medication. Given these results, the researchers conclude that there is little reason to prescribe new-generation antidepressant medications to any but the most severely depressed patients unless alternative treatments have been ineffective. In addition, the finding that extremely depressed patients are less responsive to placebo than less severely depressed patients but have similar responses to antidepressants is a potentially important insight into how patients with depression respond to antidepressants and placebos that should be investigated further.

**Additional Information.:**

Please access these Web sites via the online version of this summary at http://dx.doi.org/10.1371/journal.pmed.0050045.

- The MedlinePlus encyclopedia contains a page on depression (in English and Spanish)
- Detailed information for patients and caregivers is available on all aspects of depression (including symptoms and treatment) from the US National Institute of Medical Health and from the UK National Health Service Direct Health Encyclopedia
- MedlinePlus provides a list of links to further information on depression
- Clinical Guidance for professionals, patients, caregivers and the public is provided by the UK National Institute for Health and Clinical Excellence

## Introduction

Meta-analyses of antidepressant efficacy based on data from published trials reveal benefits that are statistically significant, but of marginal clinical significance [1]. Analyses of datasets including unpublished as well as published clinical trials reveal smaller effects that fall well below recommended criteria for clinical effectiveness. Specifically, a meta-analysis of clinical trial data submitted to the US Food and Drug Administration (FDA) revealed a mean drug–placebo difference in improvement scores of 1.80 points on the Hamilton Rating Scale of Depression (HRSD) [2], whereas the National Institute for Clinical Excellence (NICE) used a drug–placebo difference of three points as a criterion for clinical significance when establishing guidelines for the treatment of depression in the United Kingdom [1]. Mean improvement scores can obscure differences in improvement within subsets of patients. Specifically, antidepressants may be effective for severely depressed patients, but not for moderately depressed patients [1,3,4]. The purpose of the present analysis is to test that hypothesis (see Text S1 for the QUOROM checklist).

Conventional meta-analyses are often limited to published data. In the case of antidepressant medication, this limitation has been found to result in considerable reporting bias characterized by multiple publication, selective publication, and selective reporting in studies sponsored by pharmaceutical companies [5]. To avoid publication bias, we evaluated a dataset that includes the complete data from all trials of the medications, whether or not they were published. Specifically, we analyzed the data submitted to the FDA for the licensing of four new-generation antidepressants for which full data, published and unpublished, were available. As part of the licensing process, the FDA requires drug companies to report “*all* controlled studies related to each proposed indication” ([6] emphasis in original). Thus, there should be no reporting bias in the dataset we analyze.

## Methods

### Study Retrieval

Following the Freedom of Information Act (FOIA) [7], we requested from the FDA all publicly releasable information about the clinical trials for efficacy conducted for marketing approval of fluoxetine, venlafaxine, nefazodone, paroxetine, sertraline, and citalopram, the six most widely prescribed antidepressants approved between 1987 and 1999 [2], which represent all but one of the selective serotonin reuptake inhibitors (SSRIs) approved during the study period. In reply, the agency provided photocopies of the medical and statistical reviews of the sponsors' New Drug Applications. The FDA requires that information on all industry-sponsored trials be submitted as part of the approval process; hence the files sent to us by the FDA should contain information on all trials conducted prior to the approval of each medication. This strategy omits trials conducted after approval was granted.

Although sponsors are required to submit information on all trials, the FDA public disclosure did not include mean changes for nine trials that were deemed adequate and well controlled but that failed to achieve a statistically significant benefit for drug over placebo. Data for four of these trials were available from a pharmaceutical company Web site in January 2007 and were obtained from the GlaxoSmithKline clinical trial register (http://ctr.gsk.co.uk/Summary/paroxetine/studylist.asp).

We also identified published versions of the FDA trials via a PubMed literature search (from January 1985 through May 2007) using the keywords *depression*; *depressive*; *depressed*; and *placebo*; specific names of antidepressant medications; and names of investigators from the FDA trials. Potentially relevant studies were also identified through references of retrieved and review articles and from a partially overlapping list of published versions of trials submitted to the Swedish drug regulatory authority [5]. Using a standardized protocol, all retrieved abstracts and publications were compared to the FDA trials. The match between each published study and its corresponding FDA trial was independently established with 100% agreement by two investigators (BJD and a research assistant).

### Selection

**Figure 1:**
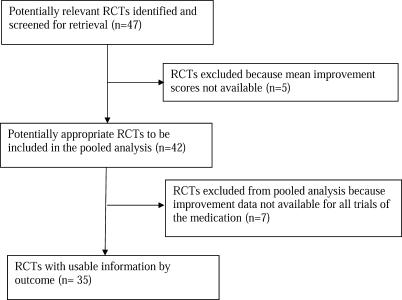
QUOROM Flow Chart

Forty-seven clinical trials were identified in the data obtained from the FDA. The trial flow is illustrated in Figure 1. Inclusion of a drug type for which unsuccessful trials were excluded biases overall results in favor of that drug type, in a way that is akin to publication bias. The purpose in using the FDA dataset is precisely to avoid this type of bias by including all trials of each medication assessed. Therefore, we present analyses only for those medications for which mean change scores on all trials were available.

### Validity Assessment

The FDA requires that rigorous standards be followed for the conduct of all efficacy trials for marketing approval [8] and also sets specific agency standards for clinical trials of antidepressant drugs [9]. In addition, the FDA independently reviews the clinical trial methods, statistical procedures, and results. The FDA dataset includes analyses of data from all patients who attended at least one evaluation visit, even if they subsequently dropped out of the trial prematurely. Results are reported from all well-controlled efficacy trials of the use of these medications for the treatment of depression. FDA medical and statistical reviewers had access to the raw data and evaluated the trials independently. The findings of the primary medical and statistical reviewers were verified by at least one other reviewer, and the analysis was also assessed by an independent advisory panel. Following FDA standards, all trials were randomized, double-blind, placebo-controlled trials. None used cross-over designs. Patients had been diagnosed as suffering from unipolar major depressive disorder using Diagnostic and Statistical Manual of Mental Disorders (DSM) criteria.

Given the above review process, we deemed it appropriate to include all studies deemed adequate and well controlled by FDA reviewers, especially as these are the data upon which the decision to approve these medications was based. Other validity criteria might yield different conclusions. In this review, some of the characteristics that may relate to the quality of trials were coded and assessed as possible moderator variables (e.g., interval of trial). The studies have similar methodological characteristics and were well controlled; therefore the methodological characteristics did not affect the final results.

### Study Characteristics

In order to generalize the findings of the clinical trial to a larger patient population, FDA reviewers sought a completion rate of 70% or better for these typically 6-wk trials. Only four of the trials reported reaching this objective, and completion rates were not reported for two trials. Attrition rates were comparable between drug and placebo groups. Of those trials for which these rates were reported, 60% of the placebo patients and 63% of the study drug patients completed a 4-, 5-, 6-, or 8-wk trial. Thirty-three trials were of 6-wk duration, six trials were 4 wk, two were 5 wk, and six were 8 wk. Patients were evaluated on a weekly basis. For this meta-analysis, the data were taken from the last visit prior to trial termination.

Thirty-nine trials focused on outpatients: three included both inpatients and outpatients, three were conducted among the elderly (including one of the trials with both inpatients and outpatients), and two were among patients hospitalized for severe depression. No trial was reported for the treatment of children or adolescents.

Replacement of patients who investigators determined were not improving after 2 wk was allowed in three fluoxetine trials and in the three sertraline trials for which data were reported. The trials also included a 1- to 2-wk washout period during which patients were given placebo, prior to random assignment. Those whose scores improved 20% or more were excluded from the study prior to random assignment. The use of other psychoactive medication was reported in 25 trials. In most trials, a chloral hydrate sedative was permitted in doses ranging from 500 mg to 2,000 mg per day. Other psychoactive medication was usually prohibited but still reported as having been taken in several trials.

### Meta-Analytic Data Synthesis

We conducted two types of data analysis, one in which each group's change was represented as a standardized mean difference (*d*), which divides change by the standard deviation of the change score (*SD*_c_) [10], and another using each study's drug and placebo groups' arithmetic mean (weighted for the inverse of the variance) as the meta-analytic “effect size” [11].

The first analysis permitted a determination of the absolute magnitude of change in both the placebo and treatment groups. Results permitted a determination of overall trends, analyses of baseline scores in relation to change, and for both types of models, tests of model specification, which assess the extent to which only sampling error remains unexplained. The results in raw metric are presented comparing both groups, but because of the variation of the *SD*_c_s, the standardized mean difference was used in moderator analyses in order to attain better-fitting models [12]. These results are compared to the criterion for clinical significance used by NICE, which is a three-point difference in Hamilton Rating Scale of Depression (HRSD) scores or a standardized mean difference (*d)* of 0.50 [1].

As known *SD*_c_s were related to mean baseline HRSD scores, these scores were used to impute missing *SD*_c_ values, taking into account both the baseline and its quadratic form and any potential interaction of these terms with group (but in fact, there was no evidence that *SD*_c_s depended on treatment group). One trial reported *SD*_c_s for its drug and placebo groups that were less than 25% the size of the other trials; because preliminary analyses also revealed that this trial was an outlier, these two standard deviations were treated as missing and imputed. In total, *SD*_c_s were known for 28 groups, could be calculated from other inferential statistics in nine comparisons (18 groups), and were imputed in 12 comparisons (24 groups) (47.38%) [13,14].

Overall analyses evaluated both random- and fixed-effects models to assess effect size magnitude; because the same trends appeared for both, for simplicity we present only the fixed-effects results. We also assumed fixed-effects assumptions in order to analyze moderators for both groups. Both *Q* [15] and *I* ^2^ [16] indices were used to assess inconsistencies from the models, not only to infer the presence or absence of homogeneity, but also (in the case of *I* ^2^) to assess the degree of inconsistencies among trials [17]. We assumed fixed-effects models in analyzing moderators using meta-regression procedures [11]. Analyses examining linear and quadratic functions for baseline levels of severity used zero-centered forms of this variable [18]. A last, mixed-effects analysis for the amount of change used a random-effects constant along with fixed-effects moderator dimensions; these models provide more conservative assessments of moderation [19].

Because the same scale was used as the primary dependent variable in all of these trials, we were also able to represent results in their original metric [11]. This form of analysis makes results more easily interpretable in terms of clinical significance because mean change scores are analyzed directly, rather than being converted into effect sizes. The analytic weights are derived from the sample size and the *SD*_c_ [11]. Finally, to show directly the amount of improvement for each study's drug group against its placebo group, we calculated the difference between the change for the drug group minus the change for the placebo group, leaving the difference in raw units and deriving its analytic weight from its standard error [11,12,20]. Analyses used these weights to examine these controlled outcomes both overall and to determine the extent to which drug-related change is a function of initial severity.

## Results

### Trial Flow

Mean improvement scores were not available in five of the 47 trials (Figure 1). Specifically, four sertraline trials involving 486 participants and one citalopram trial involving 274 participants were reported as having failed to achieve a statistically significant drug effect, without reporting mean HRSD scores. We were unable to find data from these trials on pharmaceutical company Web sites or through our search of the published literature. These omissions represent 38% of patients in sertraline trials and 23% of patients in citalopram trials. Analyses with and without inclusion of these trials found no differences in the patterns of results; similarly, the revealed patterns do not interact with drug type. The purpose of using the data obtained from the FDA was to avoid publication bias, by including unpublished as well as published trials. Inclusion of only those sertraline and citalopram trials for which means were reported to the FDA would constitute a form of reporting bias similar to publication bias and would lead to overestimation of drug–placebo differences for these drug types. Therefore, we present analyses only on data for medications for which complete clinical trials' change was reported. The dataset comprised 35 clinical trials (five of fluoxetine, six of venlafaxine, eight of nefazodone, and 16 of paroxetine) involving 5,133 patients, 3,292 of whom had been randomized to medication and 1,841 of whom had been randomized to placebo.

### Mean Change

Baseline HRSD scores, improvement, and sample sizes in drug and placebo groups for each clinical trial are reported in Table 1. As in the FDA files, studies are identified by protocol numbers. The data from these trials can be obtained from the FDA using FOIA requests and citing the medication name and protocol number. The table also includes references to published reports of the data abstracted from the FDA files, when they could be found (using the search methods described above). Studies in which data only from selected sites of a multisite study were published are not cited in the table. We have also excluded published reports in which dropouts have been removed from the data. For each of the trials, the pharmaceutical companies had submitted to the FDA data in which attrition was handled by carrying forward the last observation carried forward (LOCF) on the patient, which was the basis in all cases of the FDA review. These data and their corresponding citations appear in the table. Even in the LOCF data, there sometimes are some minor discrepancies between the published version and the version submitted to the FDA. In some cases, for example, the *N* is slightly larger in the published studies than in the data reported to the FDA. Further complicating this problem is the fact that occasionally, the company has published a trial more than once, with slight discrepancies in the data between publications. Data in the table are those reported to the FDA.

**Table 1:**
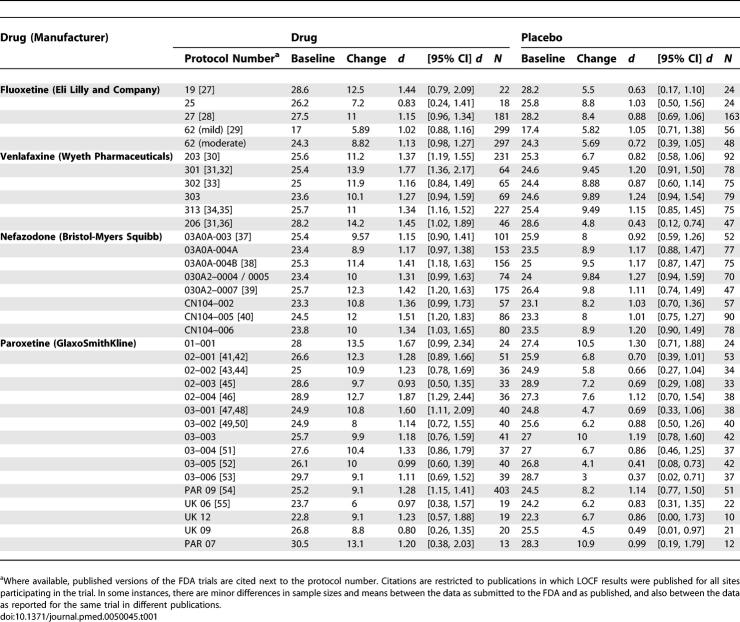
Baseline HRSD Scores, Sample Sizes, and Raw and Standardized Improvement with Confidence Intervals, as Reported to the FDA for Drug and Placebo Groups

Confirming earlier analyses [2], but with a substantially larger number of clinical trials, weighted mean improvement was 9.60 points on the HRSD in the drug groups and 7.80 in the placebo groups, yielding a mean drug–placebo difference of 1.80 on HRSD improvement scores. Although the difference between these means easily attained statistical significance (Table 2, Model 3a), it does not meet the three-point drug–placebo criterion for clinical significance used by NICE. Represented as the standardized mean difference, *d*, mean change for drug groups was 1.24 and that for placebo 0.92, both of extremely large magnitude according to conventional standards. Thus, the difference between improvement in the drug groups and improvement in the placebo groups was 0.32, which falls below the 0.50 standardized mean difference criterion that NICE suggested. The amounts of change for drug and placebo groups varied widely around their respective means, *Q*(34)s = 51.80 and 74.59, *p*-values < 0.05, and *I* ^2^s = 34.18 and 54.47. Thus, the mean change exhibited in trials provides a poor description of results, and moderator models are indicated.

**Table 2:**
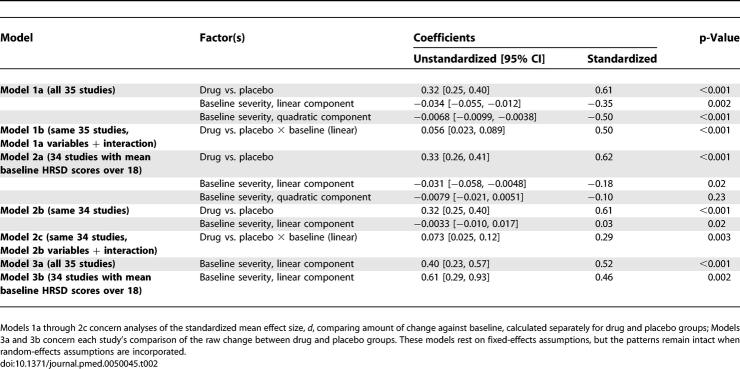
Models of Improvement in Depression Scores Based on Group Assignment (Drug versus Placebo) and Initial Depression Severity (as Gauged by HRSD)

### Drug and Initial Severity Trends in Change

**Figure 2:**
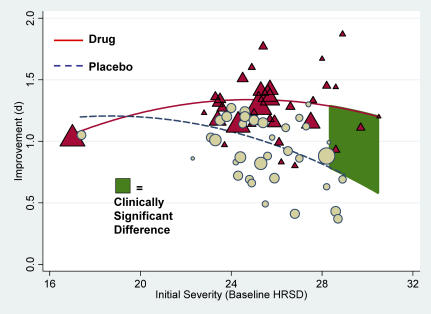
Mean Standardized Improvement as a Function of Initial Severity and Treatment Group Drug improvement is portrayed as red triangles around their solid red regression line and placebo improvement as blue circles around their dashed blue regression line; the green shaded area indicates the point at which comparisons of drug versus placebo reach the NICE clinical significance criterion of *d* = 0.50. Plotted values are sized according to their weight in analyses.

Moderator analyses examined whether drug type, duration of treatment, and baseline severity (HRSD) scores related to improvement. Although drug type and duration of treatment were unrelated to improvement, the drug versus placebo difference remained significant, and amount of improvement was a function of baseline severity (Table 2, Model 1a). Specifically, the amount of improvement depended markedly on the quadratic function of baseline severity, but the linear function of baseline severity interacted with assignment to drug versus placebo (Model 1b). Specifically, as Figure 2 shows, improvement from baseline operated as a ∩-shaped curvilinear function in relation to baseline severity, with those at the lowest and highest levels experiencing smaller gains, whereas those in-between experienced larger gains; the slope for placebo declined as severity increased, whereas the slope for drug was slightly positive. The difference between drug and placebo exceeded NICE's 0.50 standardized mean difference criterion at comparisons exceeding 28 in baseline severity. Further analyses indicated that drug type did not moderate this affect. Although venlafaxine and paroxetine had significantly (*p* < 0.001) larger weighted mean effect sizes comparing drug to placebo conditions (*d*s = 0.42 and 0.47, respectively) than fluoxetine (*d* = 0.22) or nefazodone (0.21), these differences disappeared when baseline severity was controlled.

**Figure 3:**
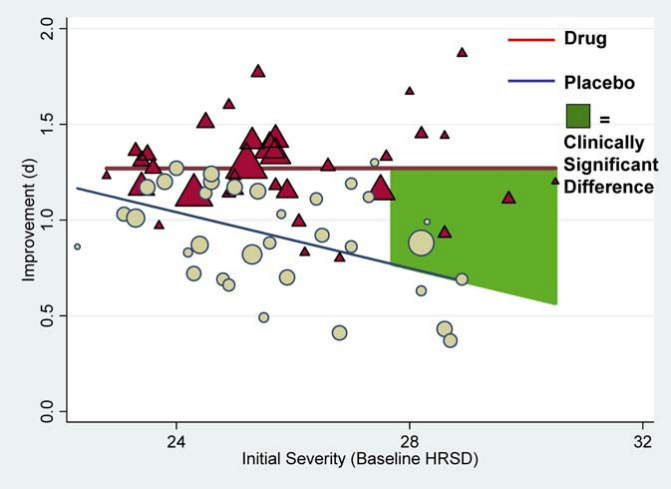
Mean Standardized Improvement as a Function of Initial Severity and Treatment Group, Including Only Trials Whose Samples Had High Initial Severity Drug improvement is portrayed as red triangles around their solid red regression line and placebo improvement as blue circles around their dashed blue regression line; the green shaded area indicates the point at which comparisons of drug versus placebo reach the NICE clinical significance criterion of *d* = 0.50. Plotted values are sized according to their weight in analyses.

For all but one sample, baseline HRSD scores were in the very severe range according to the criteria proposed by the American Psychiatric Association (APA) [21] and adopted by NICE [1]. The one exception derived from a fluoxetine trial that had two samples, one with HRSD scores in the very severe range and the other with scores in the moderate range. Because the low-HRSD condition might be considered an outlier, the analyses were performed again without it. Results continued to reveal that drug versus placebo assignment interacted with initial severity to influence improvement; yet the curvilinear function of the baseline was no longer significant, although group continued to interact with the linear component (Table 2, Model 2c). As Figure 3 shows, drug efficacy did not change as a function of initial severity, whereas placebo efficacy decreased as initial severity increased; values again exceeded NICE's 0.50 standardized mean difference criterion at comparisons greater than 28 in baseline severity. This final model comprising three simultaneous study dimensions (viz., drug vs. placebo, baseline, and the interaction) explained 51.45% of the variation in improvement. Although this model was in a formal sense incorrectly specified (*Q*_Residual_(64) = 96.07, *p* < 0.01), when a random-effects constant was instead assumed, the same pattern of results remained in this more statistically conservative mixed-effects model. A final model that incorporated even the drug types for which only some trials were available confirmed these trends.

**Figure 4:**
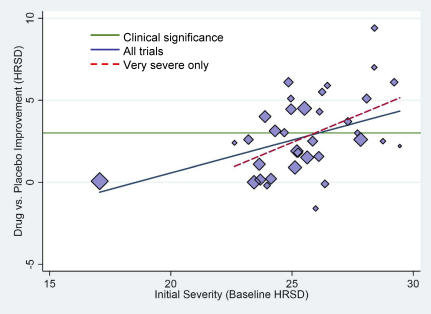
Mean Drug–Placebo Difference Scores as a Function of Initial Severity Plotted values are sized according to their sample sizes (*n*); the green line represents the NICE clinical significance criterion. The solid blue regression line represents the trend across all 35 trials; the dashed red line represents the trend excluding the left-most observation.

Figure 4 displays raw mean differences between drug and placebo as a function of initial severity, rising as a linear function of baseline severity levels (Table 2, Models 3a and 3b) even though, almost without exception, the scores were in the very severe range of the criteria proposed by APA [21]. Yet when these data are considered in conjunction with those in Figure 3, it seems clear that the increased difference is due to a decrease in improvement in placebo groups, rather than an increase in drug groups.

A visual inspection of Figure 4 suggests that studies' effects are fairly evenly distributed above and below the NICE criterion (3) but that most small studies have high baselines and show large effects. Although sample size (*N*) was negatively linked to the drug-versus-placebo differences (β = −0.34, *p* = 0.003), when mean baseline severity values are controlled, this effect disappears and the baseline effect remains significant. The interaction of sample size with baseline severity was marginally significant, *p* = 0.0586, and the pattern indicated that baseline severity was somewhat more predictive for smaller than for larger studies. Yet, because simple-slopes analyses revealed that baseline scores were significantly predictive even for the largest studies, study differences in sample size would appear to qualify neither the pattern of results we have reported nor their interpretation.

Examination of publication bias often relies on inspections of effect sizes in relation to sample size (or inverse variance) [22]. A funnel plot of the data depicted in Figure 4 indicates that the larger studies in the FDA datasets tended to show smaller drug effects than smaller studies. Although such a pattern might be construed as indicating a publication or other reporting bias, our use of complete datasets precludes this possibility, unless some small trials were not reported despite the FDA Guidelines [6]. A more plausible explanation is that trials with higher baseline scores tended to be small. In any case, funnel-plot inspections assume that there is only one population effect size that can be tracked by a comparison between drug and placebo groups, whereas the current investigation shows that these effects vary widely and that the magnitude of the difference depends on initial severity values. Consequently, funnel-plot inspection is much less appropriate in the present context. Unfortunately, there are no other tools yet available to detect publication or other reporting biases in the face of effect modifiers.

## Discussion

Using complete datasets (including unpublished data) and a substantially larger dataset of this type than has been previously reported, we find that the overall effect of new-generation antidepressant medications is below recommended criteria for clinical significance. We also find that efficacy reaches clinical significance only in trials involving the most extremely depressed patients, and that this pattern is due to a decrease in the response to placebo rather than an increase in the response to medication.

Similar to prior reports [3,4], this analysis of U.S. FDA data for four new-generation antidepressants suggests an association between initial severity and the benefit of antidepressant medication. Unlike prior studies, we restricted our analysis to complete datasets that included all trials conducted, whether published or not. Thus, simple publication bias cannot underlie the results. We compared drug–placebo differences in improvement to criteria for clinical efficacy, and we used meta-regression procedures [11] to identify the relation of severity to improvement. Although we were able to replicate previously reported decreases in the placebo response as a function of increasing baseline severity, we found no linear relation between severity and response to medication.

NICE used a three-point difference in HRSD change scores, or a standardized mean difference of 0.50, as criteria of clinical significance [1]. By that criterion, the differences between drug and placebo were not clinically significant in clinical trials involving either moderately or very severely depressed patients, but did reach the criterion for trials involving patients whose mean initial depression scores were at the upper end of the very severe depression category (mean HRSD baseline ≈ 28; Figures 2–4). Given these data, there seems little evidence to support the prescription of antidepressant medication to any but the most severely depressed patients, unless alternative treatments have failed to provide benefit.

A prior meta-analysis of published data only reported a very small significant difference between the antidepressant effect of fluoxetine and venlafaxine, but did not assess the effect of baseline severity as a moderator [23]. Our analyses failed to reveal any effect of drug type on efficacy or on the relation between severity and efficacy. It is possible that differences associated with drug type might be found with the inclusion of clinical trials conducted after the approval process, but analyses of head-to-head comparisons suggest that they are not likely to be large enough to be of clinical importance [23].

The response to placebo in these trials was exceptionally large, duplicating more than 80% of the improvement observed in the drug groups. In contrast, the effect of placebo on pain is estimated to be about 50% of the response to pain medication [24–26]. A substantial response to placebo was seen in moderately depressed groups and in groups with very severe levels of depression. It decreased somewhat, but was still substantial, in groups with the most-severe levels of depression.

Although baseline severity related to degree of improvement in the drug groups, the pattern was not linear. Instead, patients who by APA criteria were moderately depressed and those at the very high end of the severely depressed category (i.e., those with initial HRSD scores greater than 28) showed less improvement than those at the lower end of the severely depressed category. The curvilinear relation depended on only one trial of moderately depressed patients. When that outlier trial is excluded, there is no relation between baseline severity and antidepressant response. However, all of the other trials were with groups with mean initial HRSD scores in the very severe range (i.e., ≥23). What is missing from the FDA data, however, are clinical trials with patients with initial depression scores in the severe range (19–22), and there was only one study with patients in the moderately depressed range. Had groups with a wider array of baseline depression scores been assessed, the curvilinear pattern might have been more obvious; in which case, clinically significant benefits for severely depressed patients might have been obtained. To perform this task in an unbiased way, it would be necessary for data for all approved medications to be available, even those gathered after the medication is approved. Having all the information available would also obviate the need to impute missing standard deviations, a limitation of the current investigation. Public availability of complete data on approved mediations might be made a condition of approval to solve these problems.

Finally, although differences in improvement increased at higher levels of initial depression, there was a negative relation between severity and the placebo response, whereas there was no difference between those with relatively low and relatively high initial depression in their response to drug. Thus, the increased benefit for extremely depressed patients seems attributable to a decrease in responsiveness to placebo, rather than an increase in responsiveness to medication.

## Supporting Information

Text S1 QUOROM Checklist (33 KB DOC)

## Abbreviations

*d*: standardized mean difference
FDA: US Food and Drug Administration
HRSD: Hamilton Rating Scale of Depression
LOCF: last observation carried forward
NICE: National Institute for Clinical Excellence
*SD*_c_: standard deviation of the change score
