# Supplementary material for: Initial Severity and Antidepressant Benefits: A Meta-Analysis of Data Submitted to the Food and Drug Administration
Source: PLoS Med. 2008 Feb 26;5(2):e45. doi: 10.1371/journal.pmed.0050045 (PMC2253608; doi:10.1371/journal.pmed.0050045)
Supplement: Text S1 — (33 KB DOC) [file pmed.0050045.sd001.doc]

**QUORUM statement checklist: Initial Severity and Antidepressant Benefits:**

**A Meta-Analysis of Data Submitted to the FDA**

**_______________________________________________________________________________________________________________________________________________________________________________________**

**Heading Subheading Descriptor Reported? (Y/N) Heading: Subheading**

**------------------- ------------------------ ------------------------------------------------------------------------------------------------------------- --------------------------- -----------------------**

**Title**  Identify the report as a meta-analysis of RCTs Yes Title

------------------- ------------------------ ------------------------------------------------------------------------------------------------------------- --------------------------- -----------------------

**Abstract** Use a structured format^27^ Yes Abstract

**Describe**

Objectives The clinical question explicitly

Data sources The databases (ie, list) and other information sources

Review methods The selection criteria (ie, population, intervention, outcome, and study design);

methods for validity assessment, data abstraction, and study characteristics, and

quantitative data synthesis in sufficient detail to permit replication

Results Characteristics of the RCTs included and excluded; qualitative and quantitative

findings (ie, point estimates and confidence intervals); and subgroup analyses

Conclusion The main results

**------------------ ----------------------- --------------------------------------------------------------------------------------------------------------- ---------------------------- -------------------------**

**Describe**

**------------------ ----------------------- ---------------------------------------------------------------------------------------------------------------- ----------------------------- --------------------------**

**Introduction** The explicit clinical problem, biological rationale for the intervention, and rationale for review Yes Introduction

------------------ ----------------------- ---------------------------------------------------------------------------------------------------------------- ------------------------------ --------------------------

**Methods**  Searching The information sources, in detail^28^ (eg, databases, registers, personal files, expert Yes Methods: Study Retrieval

informants, agencies, hand-searching), and any restrictions (years considered, publication

status,^29^ language of publication^30,31^)

Selection The inclusion and exclusion criteria (defining population, intervention, principal Yes Methods: Selection

outcomes, and study design^32^

Validity assessment The criteria and process used (eg, masked conditions, quality assessment, and their findings^33–36^) Yes Methods: Validity Assessment

Data abstraction The process or processes used (eg, completed independently, in duplicate)^35,36^ Yes Author Contributions Study characteristics The type of study design, participants’ characteristics, details of intervention, outcome Yes Methods: Study Characteristics

definitions, &c,^37^ and how clinical heterogeneity was assessed

Quantitative data synthesis The principal measures of effect (eg, relative risk), method of combining results Yes Methods: Meta-Analytic Data

(statistical testing and confidence intervals), handling of missing data; how statistical Synthesis

heterogeneity was assessed;^38^ a rationale for any a-priori sensitivity and subgroup analyses;

and any assessment of publication bias^39^

------------------ ----------------------------- --------------------------------------------------------------------------------------------------------------- ------------------------------ --------------------------

**Results** Trial flow Provide a meta-analysis profile summarising trial flow (see figure) Yes Results – Trial Flow

Study characteristics Present descriptive data for each trial (eg, age, sample size, intervention, dose, duration,

follow-up period) Yes Results & Table 1

Quantitative data synthesis Report agreement on the selection and validity assessment; present simple summary Yes Results & Table 1

results (for each treatment group in each trial, for each primary outcome); present data

needed to calculate effect sizes and confidence intervals in intention-to-treat analyses

(eg 2X2 tables of counts, means and SDs, proportions)

--------------------- ---------------------------- ------------------------------------------------------------------------------------------------------------------- ------------------------------ -------------------------

**Discussion**  Summarise key findings; discuss clinical inferences based on internal and external validity; Yes Discussion

interpret the results in light of the totality of available evidence; describe potential

biases in the review process (eg, publication bias); and suggest a future research agenda

**________________________________________________________________________________________________________________________________________________________**
